# Sarcopenia and the Brain

**DOI:** 10.1590/0004-282X-ANP-2021-e005

**Published:** 2021-05-01

**Affiliations:** 1 Saint Louis University School of Medicine Division of Geriatric Medicine United States of America Saint Louis University, School of Medicine, Division of Geriatric Medicine, St. Louis MO, United States of America.

The term sarcopenia (“a poverty of flesh”) was first described by Irving Rosenberg in 1989. In its original format it was limited to an aging related loss of muscle that was associated with functional deterioration and earlier mortality^1^. In 2010, the European Working Group on Sarcopenia in Older Persons (EWGSOP) published a consensus definition on sarcopenia where they required both a loss of muscle mass and strength to make the diagnosis^2^. In 2019 a revised version of the EWGSOP included SARC-F as a screening test^3^. Similar consensus recommendations have been published by ICFSR and SCWD^4^^,^^5^. SARC-F is a rapid set of 5 questions developed to screen for sarcopenia^6^^,^^7^^,^^8^. There is an ICD-10 code for sarcopenia^9^.

While cognitive frailty is now a well-accepted syndrome^10^^,^^11^, sarcopenia has not been classically associated with impaired cognition. In the last few years there has been an increasing awareness of a relationship between sarcopenia and impaired cognition^12^^,^^13^ as highlighted by the article by Cipolli et al.^14^. This association between poor cognition and sarcopenia should not be surprising as 82% of frailty persons have sarcopenia^15^. This raises the question of whether poor cognition leads to sarcopenia or more importantly can loss of muscle mass lead to cognitive impairment? In addition, a number of conditions, e.g., inflammatory cytokines, diabetes mellitus, vascular disease, can cause both brain and muscle dysfunction^16^.

In the last decade there has been increasing awareness that myokines can directly affect the brain^17^. This, in part, explains the positive effects of exercise on the brain^18^. One of these myokines is Irisin, which promotes synaptic plasticity ad can improve memory in animal models^19^. Irisin is derived from fibronectin type III domain – containing protein 5 (FNDC5) and is increased with exercise^20^. Irisin is considered to play a role in exercise induced memory enhancement^21^^,^^22^. Other myokines that play a role in muscle-brain interaction include cathepsin-B, brain derived nerve growth factor insulin growth factor-1, oncostatin M, and leukemia inhibitory factor^23^^,^^24^^,^^25^. These myokines promote angiogenesis, neurogenesis, synaptic function, neuronal metabolism and autophagia.

Decline in brain function can result in a decrease in muscle function. Perhaps the most obvious example is loss of muscle and weakness following a cerebrovascular accident. Persons with memory dysfunction have a slower gait speed and a decrease in grip strength^26^^,^^27^. Persons with dementia have a deficit in “dual tasking” and tend to be less likely to exercise leading to sarcopenia. Aging itself with the reduction in axonal communication and a reduction in motor unit numbers leads to a decrease in Type 2 muscle fibers^28^.

For all of these reasons, it is not surprising that there is a relationship between sarcopenia and cognitive impairment in community dwelling older adults as found by Cipolli et al. and other studies^14^^,^^29^^,^^30^. As shown in Figure 1 there are multiple causes for the interrelationship between cognitive dysfunction and sarcopenia. It is important that these causes be recognized and treated where possible. Recently, Lundy et al.^31^ showed that the combination of cognitive stimulation therapy (CST) and exercise in older persons had a greater improvement in cognition, than CST alone. For this reason, we believe that physicians should recommend exercise therapy not only to improve muscle function but also to improve cognitive deficits.

**Figure 1:**
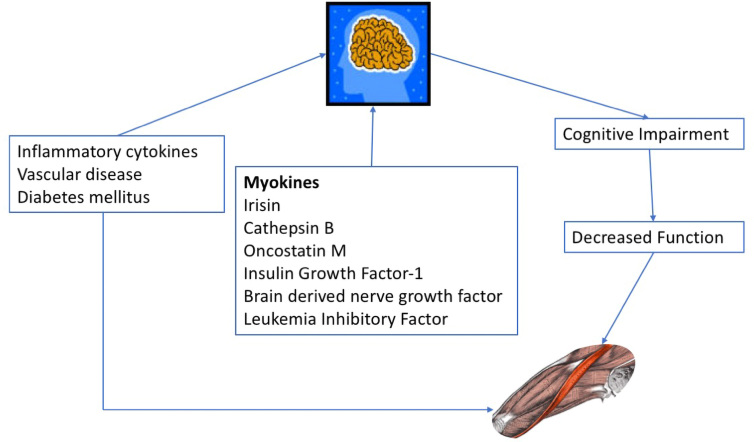
Muscle-brain interactions

## References

[1] 1. Morley JE, Baumgartner RN, Roubenoff R, Mayer J, Nair KS. Sarcopenia. J Lab Clin Med. 2001 Apr;137(4):231-43. https://doi.org/10.1067/mlc.2001.113504 10.1067/mlc.2001.113504 11283518

[2] 2. Cruz-Jentoff AJ, Baeyens JP, Bauer J, Boirie Y, Cederholm T, Landi F, et al. Sarcopenia: European consensus on definition and diagnosis: Report of the European Working Group on Sarcopenia in Older People. Age Ageing. 2010 Jul;39(4):412-23. https://doi.org/10.1093/ageing/afq034 10.1093/ageing/afq034 PMC2886201 20392703

[3] 3. Cruz-Jentoff AJ, Bahat G, Bauer J, Boirie Y, Bruyère O, Cederholm T, et al. Sarcopenia: revised European consensus on definition and diagnosis. Age Ageing. 2019 Jan;48(1):16-31. https://doi.org/10.1093/ageing/afy169 10.1093/ageing/afy169 PMC6322506 30312372

[4] 4. Dent E, Morley JE, Cruz-Jentoft AJ, Arai H, Kritchevsky SB, Guralnik J, et al. International Clinical Practice Guidelines for Sarcopenia (ICFSR): Screening, diagnosis and management. J Nutr Health Aging. 2018 Dec;22(10):1148-61. https://doi.org/10.1007/s12603-018-1139-910.1007/s12603-018-1139-930498820

[5] 5. Bauer J, Morley JE, Schols AMWJ, Ferrucci L, Cruz-Jentoft AJ, Dent E, et al. Sarcopenia: a time for action. An SCWD position paper. J Cachexia Sarcopenia Muscle. 2019 Oct;10(5):956-61. https://doi.org/10.1002/jcsm.12483 10.1002/jcsm.12483 PMC6818450 31523937

[6] 6. Malmstrom TK, Miller DK, Simonsick EM, Ferrucci L, Morley JE. SARC-F: A symptom score to predict persons with sarcopenia at risk for poor functional outcomes. J Cachexia Sarcopenia Muscle. 2016 Mar;7(1):28-36. https://doi.org/10.1002/jcsm.12048 10.1002/jcsm.12048 PMC4799853 27066316

[7] 7. Ha Y-C, Won C, Kim M, Chun K-J, Yoo J-I. SARC-F as a useful tool for screening sarcopenia in elderly patients with hip fractures. J Nutr Health Aging. 2020;24(1):78-82. https://doi.org/10.1007/s12603-019-1307-610.1007/s12603-019-1307-631886812

[8] 8. Lu J-L, Ding L-Y, Xu Q, Zhu S-Q, Xu X-Y, Hua H-X, Chen L, Xu H. Screening accuracy of SARC-F for sarcopenia in the elderly: a diagnostic meta-analysis. J Nutr Health Aging. 2021 Feb;25(2):172-82. https://doi.org/10.1007/s12603-020-1471-810.1007/s12603-020-1471-833491031

[9] 9. Anker SD, Morley JE, von Haehling S. Welcome to the ICD-10 code for sarcopenia. J Cachexia Sarcopenia Muscle. 2016 Dec;7(5):512-4. https://doi.org/10.1002/jcsm.12147 10.1002/jcsm.12147 PMC5114626 27891296

[10] 10. Tsutsumimoto K, Doi T, Nakakubo S, Kim M, Kurita S, Ishii H, Shimada H. Cognitive frailty as a risk factor for incident disability during late life; A 24-month follow-up longitudinal study. J Nutr Health Aging. 2020;24(5):494-9. https://doi.org/10.1007/s12603-020-1365-910.1007/s12603-020-1365-932346687

[11] 11. Ge M, Zhang Y, Zhao W, Yu J, Hou L, Xia X, et al. Prevalence and its associated factors of physical frailty and cognitive impairment: Findings from the West China Health and Aging Trend Study (WCHAT). J Nutr Health Aging. 2020;24(5):525-33. https://doi.org/10.1007/s12603-020-1363-y10.1007/s12603-020-1363-y32346692

[12] 12. Wearing J, Konings P, de Bie RA, Stokes M, de Bruin ED. Prevalence of probable sarcopenia in community-dwelling older Swiss people – a cross-sectional study. BMC Geriatr. 2020 Aug 26;20(1):307. https://doi.org/10.1186/s12877-020-01718-1 10.1186/s12877-020-01718-1 PMC7448475 32847545

[13] 13. Basile G, Sardella A. From cognitive to motor impairment and from sarcopenia to cognitive impairment: A bidirectional pathway towards frailty and disability. Aging Clin Exp Res. 2021 Feb;33(2):469-78. https://doi.org/10.1007/s40520-020-01550-y 10.1007/s40520-020-01550-y 32277434

[14] 14. Cipolli GC, Aprahamian I, Borim FSA, Falcao DVS, Cachioni M, Melo RC, et al. Probable sarcopenia is associated with cognitive impairment among community dwelling older adults: Results from the FIBRA study. Arq Neuro-Psyquiatr. 2021 May;79(5):XX-XX. https://doi.org/10.1590/0004-282X-ANP-2020-0186 10.1590/0004-282X-ANP-2020-0186 PMC9394561 34161525

[15] 15. Sanford AM, Morley JE, Berg-Weger M, Lundy J, Little MO, Leonard K, Malmstrom TK. High prevalence of geriatric syndromes in older adults. PLoS One. 2020 Jun;15(6):e0233857. https://doi.org/10.1371/journal.pone.0233857 10.1371/journal.pone.0233857 PMC7274399 32502177

[16] 16. Morley JE. Bidirectional communication between brain and muscle. J Nutr Health Aging 2018 Dec;22:1144-5. https://doi.org/10.1007/s12603-018-1141-210.1007/s12603-018-1141-230498818

[17] 17. Scisciola L, Fontanella RA, Rusina Cataldo V, Paolisso G, Barbieri M. Sarcopenia and cognitive function: Role of myokines in muscle brain cross-talk. Life (Basel). 2021 Feb;11(2):173. https://doi.org/10.3390/life11020173 10.3390/life11020173 PMC7926334 33672427

[18] 18. Bray NW, Pieruccini-Faria F, Bartha R, Doherty TJ, Nagamatsu LS, Montero-Odasso M. The effect of physical exercise on functional brain network connectivity in older adults with and without cognitive impairment. A systematic review. Mech Ageing Dev. 2021 Apr;196:111493. https://doi.org/10.1016/j.mad.2021.111493 10.1016/j.mad.2021.111493 33887281

[19] 19. Lourenco MV, Frozza RL, de Freitas GB, Zhang H, Kincheski GC, Ribeiro FC, et al. Exercise-linked FNDC5/Irisin rescues synaptic plasticity and memory defects in Alzheimer's models. Nat Med. 2019 Jan;25(1):165-75. https://doi.org/10.1038/s41591-018-0275-4 10.1038/s41591-018-0275-4 PMC6327967 30617325

[20] 20. de Freitas GB, Lourenco MV, De Felice FG. Protective actions of exercise-related FNDC5/Irisin in memory and Alzheimer's disease. J Neurochem. 2020 Dec;155(6):602-11. https://doi.org/10.1111/jnc.15039 10.1111/jnc.15039 32396989

[21] 21. Lourenco MV, Ribeiro FC, Sudo FK, Drummond C, Assunção N, Vanderborght B, Tovar-Moll F, Mattos P, De Felice FG, Ferreira ST. Cerebrospinal fluid irisin correlates with amyloid-β, BDNF, and cognition in Alzheimer's disease. Alzheimers Dement. 2020 Jun 21;12(1):e12034. https://doi.org/10.1002/dad2.12034 10.1002/dad2.12034 PMC7306518 32582833

[22] 22. Kim OY, Song J. The role of irisin in Alzheimer's disease. J Clin Med. 2018 Nov;7(11):407. https://doi.org/10.3390/jcm7110407 10.3390/jcm7110407 PMC6262319 30388754

[23] 23. Delezie J, Handschin C. Endocrine crosstalk between skeletal muscle and the brain. Front Neurol. 2018 Aug;9:698. https://doi.org/10.3389/fneur.2018.00698 10.3389/fneur.2018.00698 PMC6117390 30197620

[24] 24. Lee HJ, Lee JO, Lee YW, Kim SA, Seo IH, Han JA, et al. LIF, a novel myokine, protects against amyloid-beta-induced neurotoxicity via Akt-Mediated autophagy signaling in hippocampal cells. Int J Neuropsychopharmacol. 2019 Jun;22(6):402-14. https://doi.org/10.1093/ijnp/pyz016 10.1093/ijnp/pyz016 PMC6545540 31125414

[25] 25. Seok Hyung WW, Lee SG, Kim KT, Kim HS. Oncostatin M, a muscle-secreted myokine, recovers high-glucose0induced impairment of Akt phosphorylation by Fos induction in hippocampal neuron cells. Neuroreport. 2019 Aug;30(11):765-70. https://doi.org/10.1097/WNR.0000000000001271 10.1097/WNR.0000000000001271 31233447

[26] 26. Carson RG. Get a grip: Individual variations in grip strength are a marker of brain health. Neurobiol Aging. 2018 Nov;71:189-222. https://doi.org/10.1016/j.neurobiolaging.2018.07.023 10.1016/j.neurobiolaging.2018.07.023 30172220

[27] 27. Del Campo N, Payoux P, Djilali A, Delrieu J, Hoogendijk EO, Rolland Y, et al. MPI:DSA study group. Relationship of regional brain β-amyloid to gait speed. Neurology. 2016 Jan;86(1):36-43. https://doi.org/10.1212/WNL.0000000000002235 10.1212/WNL.0000000000002235 PMC4731288 26643548

[28] 28. Manini TM, Hong SL, Clark BC. Aging and muscle: a neuron's perspective. Curr Opin Clin Nutr Metab Care. 2013 Jan;16(1):21-6. https://doi.org/10.1097/MCO.0b013e32835b5880 10.1097/MCO.0b013e32835b5880 PMC3868452 23222705

[29] 29. Cipolli GC, Yassuda MS, Aprahamian I. Sarcopenia is associated with cognitive impairment in older adults: a systematic review and meta-analysis. J Nutr Health Aging. 2019;23(6):525-31. https://doi.org/10.1007/s12603-019-1188-810.1007/s12603-019-1188-831233073

[30] 30. Moon JH, Moon JH, Kim KM, Choi SH, Lim S, Park KS, et al. Sarcopenia as a predictor of future cognitive impairment in older adults. J Nutr Health Aging. 2016;20(5):496-502. https://doi.org/10.1007/s12603-015-0613-x10.1007/s12603-015-0613-x27102786

[31] 31. Lundy J, Hayden D, Pyland S, Berg-Weger M, Malmstrom TK, Morley JE. An age-friendly health system. J Am Geriatr Soc. 2018 Jan;66(1):22-4. https://doi.org/10.1111/jgs.15076 10.1111/jgs.16959 33275785

